# A Review on Lattice Defects in Graphene: Types, Generation, Effects and Regulation

**DOI:** 10.3390/mi8050163

**Published:** 2017-05-18

**Authors:** Wenchao Tian, Wenhua Li, Wenbo Yu, Xiaohan Liu

**Affiliations:** School of Electro-Mechanical Engineering, Xidian University, Number 2 Taibai South Road, Xi’an 710071, China; wctian@xidian.edu.cn (W.T.); yuwenbo@stu.xidian.edu.cn (W.Y.); xiaohanliu@stu.xidian.edu.cn (X.L.)

**Keywords:** graphene, lattice defects, vacancies, control method

## Abstract

Graphene, having a perfect two-dimensional crystal structure, has many excellent features such as a high specific surface area, and extraordinary electrical, thermal and mechanical properties. However, during the production process, lattice defects will inevitably be produced. Therefore, the performance of graphene with various defects is much lower than its theoretical value. We summarize the major advances of research into graphene defects in engineering in this paper. Firstly, the main types and causes of defects in graphene are introduced. Secondly, the influence of different defects in graphene on the chemical, electronic, magnetic and mechanical properties is discussed. Also, the control methods of graphene defects are reviewed. Finally, we propose the future challenges and prospects for the study of the defects of graphene and other nano-carbon materials.

## 1. Introduction

According to the Mermin-Wagner theory, the long-range order of two-dimensional crystal structure could be destroyed by long wavelength fluctuation [1,2]. In addition, elastic theory dictates that thin two-dimensional film exhibit instabilities (such as bending) under non-absolute temperature (i.e., >0 K) [3,4]. Based on this, it has been considered for a long time that strict two-dimensional crystals cannot exist in normal environments. However, in 2004, Novoselov et al. discovered stable monolayer graphene [5] in the laboratory. The view that “perfect two-dimensional crystal structures cannot exist steadily” was broken by the presence of monolayer graphene. Thus, a great quantity of basic studies of graphene has been intensively developed.

Graphene, a novel two-dimensional honeycomb-structured material, is formed by a single layer of sp^2^ hybrid orbital carbon atoms. Its thickness is about 0.335 nm, corresponding to the thickness of one carbon atom. Three-dimensional graphite, one dimensional nanotubes [6], and zero-dimensional fullerene [7] can be formed by graphene via wrapping, stacking, etc. Because of its unique microstructure, graphene has many excellent physical and chemical properties such as ultrahigh specific surface area, superior stiffness [8] and strength, high electron mobility, and thermal conductivity [9]. In addition, it has a semi-integer quantum Hall effect and perfect quantum tunneling effect [10]. These characteristics make graphene the most popular low-dimensional functional carbon material after fullerene and carbon nanotubes.

Due to its unique mechanical and electrical properties, graphene has numerous applications in many fields, such as micro-nano devices, reinforcing materials and thermal applications [11,12]. Graphene can also be applied to biosensing such as deoxyribonucleic acid (DNA) sequencing devices, fano resonances and glucose detection [13]. Also, it has great potential in new energy research areas such as solar cells, lithium-ion batteries and super capacitors [14].

However, the second law of thermodynamics dictates the existence of a certain amount of defects and disorders in crystalline materials. Varieties of defects in graphene are also unavoidably produced during the preparation process. Defects have a great impact on the properties of crystals and nanostructures. Different types of defects could change the topology or the curvature and then change the structure of graphene [15]. In most cases, these unavoidable defects can affect the mechanical properties as well as the thermal and electrical conductivities [16] of graphene and graphene-based nano-composites [17]. Therefore, a good understanding of the defects and impurities in graphene is useful for further improvement of graphene-based nano-engineering.

In this review, we summarize the major advances in the study of graphene defects in engineering, including the main types of graphene defects, the generation and regulation of graphene defects, and the effect of lattice defects on the properties of graphene.

## 2. Types of Defects in Graphene

Structural defects in carbon nanotubes [18,19,20,21,22] and graphite [23,24,25,26,27] are investigated in some early studies. As a result, it is not difficult to imagine that graphene should also be defective at the atomic level. In fact, it is difficult to identify accurately and quantitatively the types of structural defects contained in graphene. However, the new high-resolution transmission electron microscope (TEM) [28] provides the capability to resolve every single atom in the graphene lattice, even for suspended single-layer graphene. In addition, the scanning electron microscope (SEM) [29,30] and atomic force microscope (AFM) are widely used experimental devices for characterizing nano-materials. Therefore, theoretically predicted configurations can be directly imaged.

In general, defects in graphene can be categorized into two different groups: the first one is intrinsic defects, which is composed of non-sp^2^ orbital hybrid carbon atoms in graphene. These defects are usually caused by the existence of non-hexagonal rings surrounded by hexagonal rings; the second defects are extrinsic defects. The crystalline order is perturbed with non-carbon atoms in graphene [31].

In addition, based on previous studies on the migration of bulk crystal defects [32,33], in particular the study of the structural remodeling of carbon nanotubes under external energy disturbances [34], it is reasonable to assume that defects can be not always random and stationary, migrating with a certain mobility governed by the activation barrier and temperature [35].

### 2.1. Intrinsic Defects in Graphene

The intrinsic defects of graphene can be divided into five categories: Stone-Wales defects, single vacancy defects, multiple vacancy defects, line defects and carbon adatoms.

**Figure 1:**
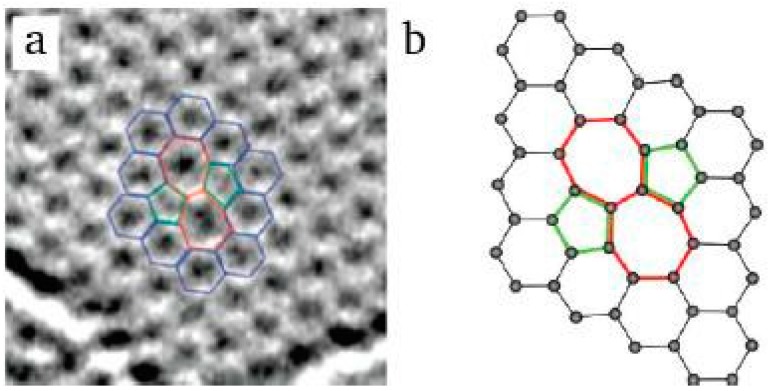
Stone-Wales defects in graphene (**a**) experimental TEM image. Reprinted with permission from [37]; copyright (2008) American Chemical Society; (**b**) atomic structure as obtained from density functional theory (DFT) calculations. Reprinted with permission from [38]; copyright (2010) American Chemical Society.

**Stone-Wales Defects**: The Stone-Wales defects of graphene are created by the rotation of a single pair of carbon atoms, thus resulting in adjacent pairs of pentagonal and heptagonal rings. Therefore, the formation of the defects does not result in any introduction or removal of carbon atoms or dangling bonds. The formation energy of this defect can be about 5 eV [35,36]. Stone-Wales defects can be introduced intentionally using electron radiation or rapid cooling in high-temperature environments. Figure 1 shows the TEM image of Stone-Wales defect and the calculated atomic structure [37,38]. The reason for the formation of these defects may be electron impact.

**Figure 2:**
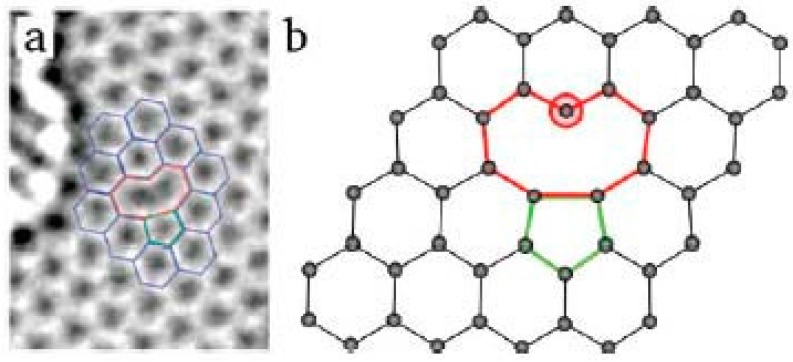
Single vacancy defect in graphene: (**a**) experimental TEM image. Reprinted with permission from [37]; copyright (2008) American Chemical Society; (**b**) atomic structure as obtained from DFT calculations. Reprinted with permission from [38]; copyright (2010) American Chemical Society.

**Single vacancy defects**: If a single carbon atom is missed in a carbon hexagon ring, there will be a single vacancy in graphene. Figure 2 shows the TEM image of the single vacancy defect and the calculated atomic structure [37,38]. The graphene undergoes a Jahn-Teller distortion to minimize the total energy. Two of the three dangling bonds are connected to each other and towards the missing atom. One dangling bond remains, owing to geometrical reasons. The formation of the vacancy defect with such a dangling bond requires a higher energy than the Stone-Wales defect. Indeed, calculations have given the value of formation energy *E_f_* ≈ 7.5 eV [19,23].

**Figure 3:**
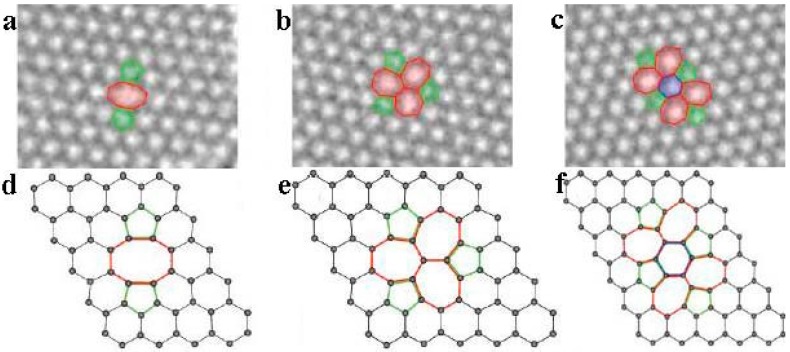
Multiply vacancy defects in graphene: (**a**–**c**) experimental TEM images (**d**–**f**) atomic structures as obtained from DFT calculations. Reprinted with permission from [38]; copyright (2010) American Chemical Society.

**Multiple vacancy defects**: Based on single vacancy defects, if there is another loss of a carbon atom, it will be a double vacancy defect. Figure 3 shows the TEM image and atomic arrangement structure diagram of three different observed multiple vacancy defects [38]. As shown in Figure 3a,d, there are two pentagons and one octagon with no dangling bond, instead of four hexagons. The simulation results show that the formation energy of this double vacancy defect can be about 8 eV [19]. The theoretical calculations show that the defect of Figure 3a,d can be transformed into the defect of Figure 3b,e under certain conditions. Furthermore, the latter one is more likely to be created because of its lower formation energy (about 7 eV). In addition, experiments also proved that the probability of this defect is indeed higher than the former one. Similar to the creation of a Stone-Wales defect, the defects shown in Figure 3b,e undergo a rotation of C–C bonds. Obviously, this rotation reduces the total energy of the graphene structure and makes the whole system more stable. The formation energy of the defect shown in Figure 3c,f is between those of the first and second vacancy defects as mentioned [38]. One step further would be the transformation of the second vacancy defect into more complicated vacancy defects (Figure 3c) by rotating another bond. The removal of more carbon atoms may result in larger and more complex defect reconstructions.

**Figure 4:**
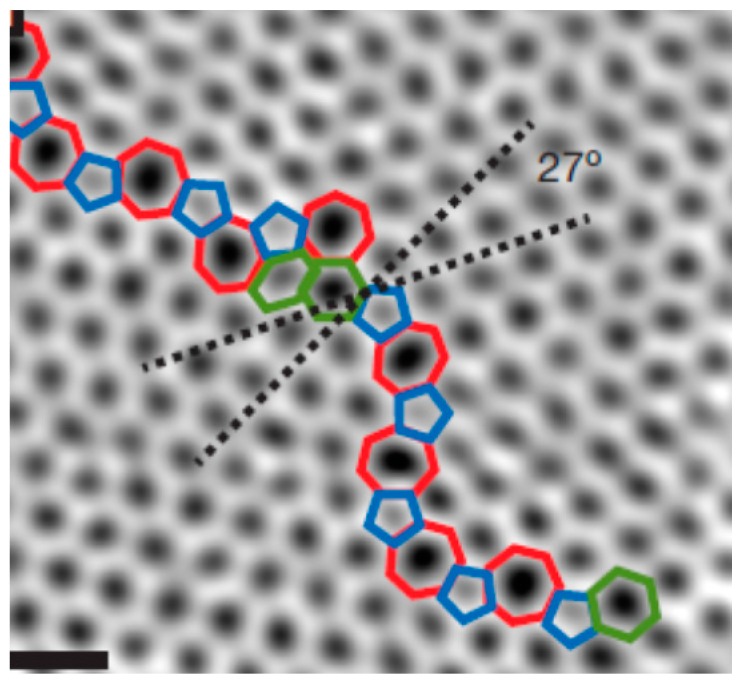
Aberration-corrected annular dark-field scanning transmission electron microscopy (ADF-STEM) of line defects in graphene; scale bars: 5 Å. Reprinted with permission from [40]; copyright (2010) Nature Publishing Group.

**Line Defects**: During the process of preparing graphene by chemical vapor deposition (CVD) [39], graphene begins to grow at different positions on the metal surface. The CVD method makes graphene polycrystallinity almost unavoidable. The randomness of growth leads to different crystallographic orientations in different locations. When the graphene grows to a certain size, cross-fusion begins. Figure 4 shows the graphene line defect [40]. As highlighted in Figure 4, the two crystals are stitched together predominantly by a chain of pentagons, heptagons and hexagons. The grain boundary is not straight, and the defects along the boundary are not periodic. Also, similar line defects in graphene have also been observed many times in other studies [41,42,43].

**Figure 5:**
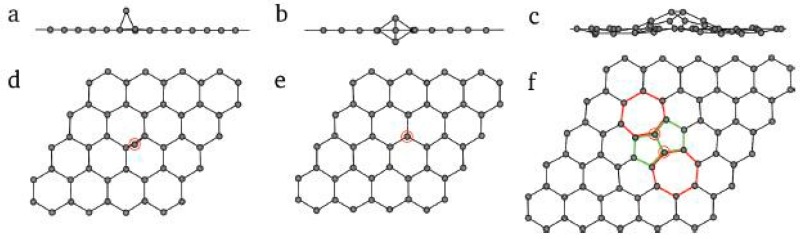
The introduction of defects to the outside surface of carbon atoms in graphene: (**a**–**c**) space structure (**d**–**f**) the location of the introduced carbon atom. Reprinted with permission from [38]; copyright (2010) American Chemical Society.

**Out-of-plane carbon adatoms**: The missing carbon atoms, which are generated from single and multiple vacancy defects, may be not completely divorced from the graphene plane. Rather, these carbon atoms migrate on the surface of the graphene after separating from the original carbon hexagon ring. When the carbon atoms migrate to another new in-plane position, a new bond is formed. If carbon atoms interact with a perfect graphene layer, new defects may be caused. Such defects will destroy the original planar structure and form a three-dimensional structure. Figure 5 shows three typical out-of-plane carbon adatoms. As shown in Figure 5a,d, carbon adatom and graphene layer form a bridge configuration (on top of a C–C bond). Figure 5b,e exhibits the metastable dumbbell configuration caused by a carbon atom migrating through the lattice. Figure 5c,f shows the inverse Stone-Wales defect formed by two migrating carbon adatoms. The out-of-plane carbon atoms have a very fast migration rate or high formation energy. Therefore, it is difficult to capture them through various microscopic techniques such as TEM, STM, etc. There is no relevant research about experimental observation of out-of-plane carbon adatoms. However, based on the earlier studies on the activation mechanism of activated carbon, carbon and oxygen atoms can migrate on the surface of carbon layer [44]. Therefore, the existence of out-of-plane carbon adatoms can be confirmed. In fact, this defect should have a variety of spatial configurations. In addition, the structure tends to be complicated as the number of introduced carbon atoms increases [45]. The out-of-plane carbon adatom undoubtedly destroys the two-dimensional crystal form of graphene. In particular, some defects (Figure 5a) change the hybridization of the carbon atoms in the layer. Some degree of sp^3^-hybridization can appear locally. A feasibility study using such defects is currently being carried out [46,47]. Obviously, it is a big challenge to make such defects controllable.

### 2.2. The Introduction of Defects in Graphene

The introduced defects of graphene can be divided into two categories: foreign adatoms and substitutional impurities. These two types of defects are described below.

**Foreign adatoms**: In the case of CVD or strong oxidation method, metal atoms or oxygen-containing functional groups are inevitably introduced to the surface of graphene during the process. These adatoms are bonded with the nearest carbon atoms with covalent bonding or weak van der Waals interaction. Such kinds of defect are called foreign adatoms. Recent studies have shown that metal adatoms can lead to significant migration on the surface of graphene [48]. Based on the experimental analysis, there are many theoretical studies on the defects of foreign adatoms on graphene. Some of these studies have focused on adsorption and surface motion [49], while others have investigated the relationship between electrical properties, magnetic properties and the defect [50]. In general, such foreign adatoms are oxygen atoms or oxygen-containing functional groups such as a hydroxyl groups or a carboxyl groups. This defect comes from a kind of graphene preparation method—the Hummers method. This method is derived from the study of Hummers for the preparation of oxidized graphite. Although many researchers have improved this method for graphene [51], the basic process is similar. Strong oxidants are used throughout the process, such as concentrated sulfuric acid, nitric acid, and potassium permanganate, etc. The graphite sheet is exfoliated and oxidized under the action of strong oxidizing agent, and then prepared with a reduction method such as thermal or chemical reduction [52,53]. A reduction agent is used to eliminate oxygen-containing functional groups. Finally, graphene has been prepared with this method [54].

In fact, the oxygen atoms in graphene are difficult to completely remove during the subsequent reduction process. Whether thermal reduction or a reducing agent is used, the final preparation of graphene will always contain a certain amount of residual oxygen atoms. Also, the oxygen content and the existence form can be characterized by photoelectron spectroscopy [55].

**Figure 6:**
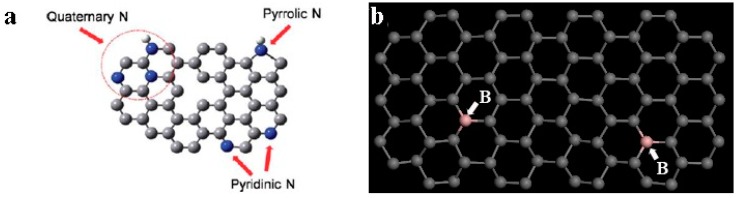
Graphene in-plane heteroatom substitution defect model: (**a**) nitrogen defects (**b**) boron defects. Reprinted with permission from [56]; copyright (2010) American Chemical Society.

**Substitutional impurities**: Some atoms such as nitrogen, boron, etc., can form three chemical bonds and so can replace the carbon atoms in graphene. These heteroatoms constitute graphene substitutional impurity defects. Figure 6 shows a graphene molecular structure model with such a defect. Nitrogen and boron atoms can exist independently in graphene. Also, they can exist simultaneously by method control [56].

In fact, nitrogen atoms and boron atoms are deliberately introduced into the graphene by method control. The reason for this is that nitrogen-doped and boron-doped graphene has excellent properties in terms of catalytic activity and conductivity [57]. Meanwhile, its conductivity and other properties are also excellent. The introduction of nitrogen and boron changes the electron cloud around graphene in the local area. In addition, this makes these regions more active [58]. Of course, as with substitutional impurities, nitrogen and boron atoms themselves have their own unique properties which also change the characteristics of graphene.

### 2.3. Double Graphene Structure Defects

When constructed in a layered manner, the graphene forms a graphite-like structure. If the graphene itself is free of defects, there will be no chemically bonded carbon atoms between the layers. However, since there are intrinsic defects (holes, dangling bonds or carbon atoms in the migrating state) in the graphene, even if only two layers of graphene are laminated, the defective graphene sheet layers will form new chemical bonds with adjacent carbon atoms [59].

If the stacking process involves more layers of graphene, the structural defects will be more complex. These complex defects are then likely to ultimately affect the macrostructure of the building material. In addition, it will affect the physical and chemical properties of the material. Monolithic graphene and graphene nanosheets are not infinitely large in space scale, so graphene in different stacking regions must involve concurrent domain processes in the construction of the graphite structure. If the domain processes are not good, it will result in a lack of long-range order in the material. This will also cause material defects [60].

## 3. Preparation of Graphene and Generation of Defects

The preparation method of graphene has a significant relationship with the formation of defects. Generally speaking, the introduction of defects in graphene cannot be avoided in the preparation process, due to the preparation method, temperature, environment and so on. The main defects are morphological and chemical-doped defects. The most common methods of preparation can be divided into two categories: CVD and graphene exfoliation. In addition, different preparation methods introduce various defects. For CVD methods, topological defects are often introduced, mainly because the dissolution technique is not mature enough. During the separation process, the structure of graphene will sustain damage, such as regional overlapping or rupture. Furthermore, the choice of substrate also has a certain impact on graphene defects. Commonly used substrates are Cu, Si and Ni substrate. These defects will have a great impact on the performance of graphene, so that its performance in reality is far below its theoretical value. However, CVD can produce a large area of graphene, so it has a certain industrial and engineering value.

Another method is graphene exfoliation. This type of method will inevitably lead to defects, but compared with CVD, defects result mainly from the oxidation and reduction process [61]. Various oxidizing agents and temperatures will have an impact on graphene. Also, due to the uneven reaction, the product is not all single-structural graphene oxide, but is also mixed with some graphite oxide or multi-structural graphene oxide. In addition, these oxides are often bonded with different functional groups, such as alcohol and aldehyde groups. Furthermore, whether it is a chemical or thermal process, varying degrees of chemical defects will be introduced. The product is called reduced graphene oxide. Some functional groups still remain after the reaction. One interesting thing is that the oxidation method can open the carbon nanotubes, so that three dimensions reduces to two dimensions [62]. However, the product often consists of uneven layers, resulting in structural defects.

In the previous section, we discussed the defect structure of graphene, and also analyzed some causes of these defects. In this section, generation of these defects will be categorized. In summary, there are three mechanisms which can lead to nonequilibrium defects in graphene: irradiation with particles, chemical treatment and crystal growth.

**Particle irradiation**: As mentioned above, when the electron beam with the appropriate energy bombards the surface of the graphene, the carbon atoms of the graphene will leave their lattice sites due to the energy interaction. These carbon atoms will be sputtered away or get adsorbed onto the sheet and migrate on the surface of the graphene. This can result in or form new defects. It is not difficult to understand that since the electron beam can disengage the carbon atom from its original position in the graphene, it can also act on the heteroatom, causing adatom defects in the graphene. At present, the reduction reaction of oxidized graphene by electron beams has been studied [63]. Of course, not only the electron beam, but also the ion beam, γ-ray can be used for defect production in graphene.

**Chemical treatment**: In order to prepare or modify graphene, graphene is treated with a chemical reagent containing oxygen, nitrogen, boron and other elements. These treatments inevitably introduce heteroatom defects into graphene. In some cases, these defects are inevitably introduced due to the limitations of process route (such as the Hummers method) [64]. Also, some defects are deliberately introduced for the purpose of modifying graphene (such as the introduction of nitrogen atoms or boron atoms into graphene to improve the graphene properties).

**Crystal growth**: Large-scale, low-defective graphene can be produced by the CVD method [65]. In a large amount of preparation methods, the graphene prepared by the CVD method shows relatively few defects in the Raman spectroscopy test. Therefore, it is a promising preparation method. However, during the CVD preparation process, the growth of a graphene layer does not normally occur slowly atom-by-atom from one nucleus, but rather as a relaxation of a mental-carbon system with many nuclei [66]. Then, the different crystal orientations lead to graphene line defects. The length of this defect is longer, so that the prepared graphene cannot become the uniform defect-free two-dimensional crystal on a large scale. It should also be noted that, although the CVD method can produce low-defective graphene, stripping graphene completely from the metal substrate represents a great challenge.

## 4. Effect of Defects on Properties of Graphene

It is well known that graphene has many unique physical and chemical properties because of its perfect and unique hexagonal single-layer carbon atom flake structure. So when it comes to lattice defects, it is very easy to think of its negative impact. For example, the actual conductivity of prepared graphene is usually lower than the theoretical calculation value. The result of this is that the existence of defects destroys the geometric symmetry and perfection of the graphene nanobelt. On one hand, lattice defects break the integrity and symmetry of graphene crystals and accordingly influence electronic transmission. On the other hand, the gap formed by the defect opens the ion transport path. Also, it enhances interaction between atoms and graphene. It contributes to the application of graphene-based composites. In the following, we will give an objective and comprehensive review of the effects of various lattice defects on the macroscopic properties of graphene.

### 4.1. Magnetic Properties

Although ideal graphene is not a magnetic material, defective graphene has shown a response signal to magnetic fields [67]. This has attracted considerable interest. Wang Yan et al. studied the hysteresis curve of graphene oxide and graphene materials prepared by high temperature reduction [68]. It was found that, unlike graphene oxide, oxidized graphene reduced at 400 °C and 600 °C exhibits ferromagnetism at room temperature. It is believed that such ferromagnetism is caused by the intrinsic defects formed by the removal of oxygen-containing functional groups from oxidized graphene at high temperatures. The new intrinsic defects in the reduction reaction of graphene have been studied by some research [68]. Also, the presence of defects leads to ferromagnetism in graphene [69]. Obviously, it remains to be studied what kinds of specific intrinsic defects are created in high-temperature reduction, and how these defects specifically affect the magnetic properties of graphene. This is also reflected in the research of Wang Yan et al.: reduced oxidized graphene does not have ferromagnetism at a temperature of 800 °C, and does not meet the magnetic law at 400 °C or 600 °C [68]. Sepioni et al. found that graphene has no ferromagnetism in the temperature range of 2–300 K [70]. It seems that the conclusions of several researchers are contradictory. In fact, if we carefully compare the process, it is not difficult to find that Wang Yan et al. used reduced graphene to do the test, while the graphene used in the study of Sepioni et al. was prepared by solvent sonication. It is clear that graphene prepared by two different methods is unlikely to be comparable in terms of two-dimensional scale or three-dimensional thickness, and especially in terms of the types of crystal defects. Therefore, the question of how to find a unified mechanism for the relationship between the magnetic properties and defects of graphene remains a challenge.

### 4.2. Electrical Properties

Graphene defects change the bond length of the interatomic valence bond. They also change the type of the hybrid trajectories of the partial carbon atoms. The changes of bond length and orbital make the electrical properties of the graphene defect domain change.

Graphene point defects and single vacancy defects form an electron scattering center on the surface of graphene. This center affects electron transfer [71], resulting in a decrease in the conductivity of graphene. In current methods for the preparation of graphene, point and single vacancy defects are unavoidable, which explains why the actual conductivity of graphene is different from the ideal state. Furthermore, it also indicates a direction for subsequent study: reducing the intrinsic defects of graphene to improve its conductivity.

Compared with intrinsic defects, the effect of the foreign atoms defects on the electrical properties of graphene is more complicated and interesting. Studies have shown that graphene oxide is not a conductive material and its square resistance can reach more than 10^12^ Ω [72]. It is speculated that graphene conductivity decreases because of the existence of oxygen atoms and oxygen-containing functional groups. However, other theoretical studies have pointed out that oxygen atom defects on graphene, such as C–O–C defects, may make the graphene support metal conductive if the position is reasonable [73].

Different from the introduction of oxygen atoms, a large number of studies have pointed out that in-plane foreign atom defects formed by nitrogen and boron atoms can improve the conductivity of graphene. Biel et al. have shown that the nitrogen and boron atoms cause resonance scattering on graphene, which affects the electrical properties of grapheme. Further studies also show that the position of nitrogen and boron atoms, the two-dimensional width of graphene and its own symmetry will affect the electrical properties of graphene [74].

The electronic properties of graphene are strongly affected by the adsorption of molecules, which makes this material very attractive for gas sensing applications. In this case, graphene is widely used in gas sensors for detecting gas concentration. In addition, the structure and electronic properties of graphene-molecule adsorption adducts are strongly dependent on the graphene structure and the molecular adsorption configuration. Research indicates that defective graphene shows the highest adsorption energy with CO, NO and NO_2_ molecules.

### 4.3. Mechanical Properties

The theoretical Young’s modulus of graphene can reach as high as 0.7–1 TPa, but different defects will affect the modulus. Hao Feng et al. investigated the effects of point defects and single vacancy defects on mechanical strength [75]. They found that the Young’s modulus of graphene decreased as the density of two defects rose. The relationship between the density of single vacancy defects and the percentage change of Young’s modulus (the Young’s modulus of defective graphene/non-defective graphene) is linear. However, the relationship between point defect density and the Young’s modulus is nonlinear. Furthermore, with the increase of density, the Young’s modulus change rate gradually represents a platform. This means that the Young’s modulus is not sensitive to point defect density. Zandiatashbar et al. also studied the mechanical properties of the graphene under the presence of sp^3^ hybrid carbon atoms and vacancy defects. It was found that the elastic modulus of the graphene was insensitive to defect density with sp^3^ hybrid carbon atoms. In contrast, vacancy defects present quite the opposite case. Vacancy defects will produce a significant reduction in the elastic modulus of graphene [8].

Studies into the effect of extrinsic defects on the mechanical properties of graphene are also underway. It was found that Young’s modulus with C–O–C heteroatom defects was 42.4% lower than that of non-defective graphene. But the tensile strength was almost unchanged. This phenomenon was due to the introduction of oxygen atoms. The foreign oxygen atoms cause the bending of the graphene sheet. The deformation of graphene increases after applying loading. The tensile strength of graphene depends on the strength of the C–C bond. The two carbon atoms connected to the oxygen atom itself are still connected to each other. Therefore, even if C–O–C defects exist, the change of graphene’s tensile strength is small [76]. According to the above study, it is not difficult to find that the intrinsic defects of graphene, especially vacancy defects, greatly affect the tensile strength of graphene, while extrinsic defects only influence graphene’s deformation modulus.

### 4.4. Thermal Conductivity Properties

Edeal graphene has a large thermal conductivity of about 5000 W/m·K [77]. Defects will change the thermal conductivity. For example, if there is a point defect or a single vacancy defect in the graphene, the thermal conductivity will rapidly decrease to 20% of the former figure. When the defect concentration further increases, the thermal conductivity drops slowly. The cause of this change is that, when the concentration of graphene is low, the defects will become the center of heat flow scattering. This center weakens the heat dissipation potential of graphene [78].

The influence of extrinsic defects on the thermal conductivity of graphene can also be studied by molecular dynamics simulation. For example, the study found that when some of the carbon atoms on the graphene become sp^3^ hybridized, assuming that the carbon atoms connected to the other three carbon atoms and one hydrogen atom, the foreign hydrogen defects will result in a decrease in thermal conductivity. Even if this defect is introduced into 2.5% of carbon atoms in graphene, the thermal conductivity of graphene will be reduced by 40%. Further studies have shown that the random dispersion of hydrogen atoms will greatly influence the thermal conductivity of graphene [79].

### 4.5. Chemical Properties

The study of the relationship between defects and the chemical properties of graphene has focused on the introduction of extrinsic defects into the graphene. This may be due to the fact that graphene without heteroatom defects is chemically inert, even if it contains intrinsic defects. Therefore, it is not easy to carry out chemical reactions.

It has been previously discussed that the introduced heteroatoms such as nitrogen will result in a higher activity of graphene. As a result, it can be used in catalytic catalyst [80] and lithium ion battery fields [81]. In addition, studies have shown that the introduction of boron atoms may also alter the graphene’s absorbency of light [82]. This is likely to bring new directions for the research of graphene in the field of photocatalysis.

With regard to the chemical application prospects raised by extrinsic defects in graphene, most attention has been paid to the introduction of oxygen atoms. The reason for this research trend can be summed up in two points: (1) Oxidized graphene carries some oxygen-containing groups such as hydroxyl and carboxyl. It makes oxidized graphene hydrophilic, so that it disperses uniformly in water. Oxidized graphene and many salt compounds or hydrophilic polymers form hydrogen bonds or ionic bond interactions, so these substances can be uniformly loaded on graphene in water. Such composite materials can be applied in catalytic [81,83], lithium [84,85,86], super capacitor [87,88], drug introduction [89] and other fields; and (2) Oxidized graphene has good self-assembly and film-forming properties. Thus, oxidized graphene has good application prospects in the flexible film field.

## 5. Regulation of Graphene Defects

Graphene defects are closely related to their properties. In order to let graphene have better characteristics and achieve specific purposes, we need to regulate and control graphene defects. In the control methods, some aim to increase the graphene activity or to tailor graphene to manufacture micro-nano devices. Others aim to improve the ordering of graphene crystal structure.

### 5.1. Defects Manufacturing

Compared with intrinsic defects, extrinsic defects are more convenient to be produced experimentally. The introduction of extrinsic defects can be brought about through chemical reactions. As long as the chemical reagents are active enough under certain conditions, other heteroatoms can be introduced into the graphene. The most typical method is the introduction of an oxygen-containing functional group into the graphene, i.e., the preparation of graphene oxide [90]. Moreover, nitrogen-doped defects can be introduced into graphene by a plasma atmosphere [57]. Boron-doped defects can be introduced into graphene through chemical reactions between the graphene and compound containing boron under high temperatures [58].

Of course, the study of the manufacture of intrinsic defects in graphene is also developing. There are two main approaches. One is the use of high-energy particle beam bombardment. This can be used on the graphene surface, which gives carbon atoms enough energy to escape from lattice sites. For example, the use of an argon ion beam can tailor vacancy defects on graphene. This method has good prospects in constructing graphene nanodevices [91]. The other is through the use of chemical methods. First, catalytic metal atoms should be loaded onto the graphene surface. Then, the metal atoms catalyze the carbon atoms on the graphene. They make carbon atoms spill from graphene in the gas phase. Thereby, intrinsic defects will be created. For example, platinum atoms are loaded on the graphene, and are then heated to 1000 °C in a hydrogen atmosphere [92]. The platinum-catalyzed carbon atoms react with hydrogen to form methane. The graphene obtained by this method displays high adsorption activity because of the presence of dangling bonds in the defects domain. This is expected to be used as a carbon dioxide storage material [93].

### 5.2. Defects Reduction

The reduction of defects in graphene can be considered from two perspectives. One is to use the appropriate preparation method to decrease defect concentration; the other is to repair graphene that contains defects.

Nowadays, graphene with few defects can be prepared by crystal growth on the metal surface. For this method, metals that can be used include Co [94], Ru [95], Pt [96,97], Ir [41], Cu [92], Ni [98] and so on. A gaseous hydrocarbon such as methane, ethylene, acetylene or a mixture of the above-mentioned gasses and hydrogen is generally selected as the carbon source. Previously, application of this method was limited because the carbon source could only be gas. In 2010, the use of solid carbon sources on the Cu surface for preparation was reported [99]. This provided new ideas for practicality. The specific operation includes the following steps: first, coat polymethyl methacrylate on the surface of Cu, and then leave in an atmosphere composed of H-Ar mixed gas at 800–1000 °C for 10 min. Graphene can then grow on the metal surface. Therefore, graphene prepared by this method exhibits a very low defect peak intensity in the Raman spectrum. This is a very promising graphene defect control program. However, identifying how to strip the graphene film from the metal surface without damage is a big challenge. In addition, recent studies have found that the use of ultrasound techniques in the liquid phase of peeling graphite can obtain low-defective graphene. With the development of the research in this area, the production of graphene by this method is increasing. Also, it is expected to produce low defect graphene in large quantities.

Although the above methods can reduce the defects of oxygen extrinsic atoms, the removal of external defects is often accompanied by the loss of carbon atoms. This will result in intrinsic defects. In Raman spectroscopy, the ratio of *I*_D_ (the peak intensity of the defective structure) to *I*_G_ (the peak intensity of the regular graphene structure) does not change or even increase. A recent study solves this problem by placing the graphene oxide into plasma methane [100]. This is a method for producing large-scale graphene in the future. As a result, there are a variety of repair methods that are worth exploring.

## 6. Conclusions

Due to its excellent physicochemical properties, graphene plays a significant role in today’s nanoscience. A variety of defects, including vacancies, interstitials, topological defects and line defects are unavoidable in experiments. The defects of graphene have a direct impact on the properties of the graphene. Although a large number of experiments or theoretical simulations have been carried out for the defects of graphene, based on the wide application prospect of graphene materials, the study of its defect characteristics needs further enrichment.
